# Metformin Toxicity Leading to Severe Refractory Hyperkalemia and Metabolic Acidosis: A Case Report

**DOI:** 10.7759/cureus.63130

**Published:** 2024-06-25

**Authors:** Laxman Wagle, Dhiraj R Regmi, Rashmita Regmi, Sishir Poudel, Hom Nath Pant

**Affiliations:** 1 Internal Medicine, Ascension Saint Agnes Hospital, Baltimore, USA; 2 Internal Medicine, Nepalese Army Institute of Health Sciences, Kathmandu, NPL; 3 Nursing, Karnali Academy of Health Science, Jumla, NPL; 4 Internal Medicine, BP Koirala Institute of Health Sciences, Dharan, NPL

**Keywords:** acute kidney injury, metformin therapy, diabetes type 2, refractory hyperkalemia, metformin induced lactic acidosis

## Abstract

Metformin is a widely prescribed, oral, anti-diabetic agent for the treatment of type 2 diabetes mellitus (DM2). While generally well-tolerated, metformin can accumulate in patients with acute kidney injury (AKI) or chronic kidney disease (CKD), leading to potentially life-threatening complications such as metformin-associated lactic acidosis (MALA). Severe hyperkalemia is a rare but serious manifestation of metformin toxicity.

We report a case of a 74-year-old African American man with DM2, hypertension, and CKD stage 3a, who presented with nausea, vomiting, lethargy, and diarrhea. Laboratory findings revealed severe AKI with a creatinine level of 8 mg/dL (baseline 1.7 mg/dL) and a potassium level of 7.8 mEq/L. The patient developed refractory hyperkalemia requiring multiple interventions and eventually continuous renal replacement therapy. Further evaluation revealed metformin-induced severe lactic acidosis with a metformin level of 21 mcg/mL (therapeutic range <5 mcg/mL). This case highlights the importance of recognizing metformin toxicity as a potential cause of severe, refractory hyperkalemia and metabolic acidosis in patients with AKI or CKD. Early recognition and prompt discontinuation of metformin, along with appropriate management of electrolyte disturbances and metabolic derangements, are crucial in preventing life-threatening complications.

## Introduction

Metformin is a first-line, oral, anti-diabetic agent used in the management of type 2 diabetes mellitus (DM2). Metformin has a considerable body of evidence that has been accumulated over the last 30 years, demonstrating its safety and tolerability along with a paucity of long-term side effects [1]. Patients on long-term therapeutic doses of metformin may develop toxicity when there is an acute deterioration of their renal function. This can manifest as metformin-associated lactic acidosis [2]. Although hyperkalemia is a common electrolyte disturbance in patients of chronic kidney disease (CKD) [3], there are no case reports in the literature documenting severe refractory hyperkalemia in the setting of metformin use. Here, we describe a case of a 74-year-old man with a history of type 2 diabetes mellitus (DM2), hypertension, and CKD stage 3a, who presented with lactic acidosis and severe refractory hyperkalemia in the setting of metformin therapy for DM2.

## Case presentation

A 74-year-old African American male, with a medical history of type 2 diabetes mellitus (DM2), hypertension, and stage 3A chronic kidney disease (CKD), presented with a two-week history of nausea and vomiting. He also reported poor appetite, lethargy, and diarrhea over the past two days. Additionally, his urine output had decreased over the past week. The patient denied chest pain, shortness of breath, palpitations, edema, abdominal pain, bloody emesis, or bloody stools. His current medications included metformin 1500 milligram (mg), aspirin 81 mg, atorvastatin 80 mg, and doxazosin 4 mg daily. He had no history of smoking, alcohol, or illicit drug use.

Upon admission, vitals showed a temperature of 36.5 degrees Celsius, heart rate of 61 beats per minute, blood pressure of 125/57 mmHg, respiratory rate of 30 breaths per minute, and oxygen saturation of 98 percent on 2 liters of oxygen via nasal cannula. The patient was alert, and oriented but appeared ill. The physical examination was otherwise unremarkable. Initial laboratory tests revealed a white blood cell (WBC) count of 7.7 thousand per microliter (K/µL), hemoglobin of 7.4 gram per deciliter (g/dl), and platelet count of 248 K/µL. The basic metabolic profile showing potassium, bicarbonate, and creatinine is given in Table 1. Venous blood gas showed pH 7.05, partial pressure of carbon dioxide (pCO2) 22 mm hg, bicarbonate (HCO3) 6 milliequivalent per liter (mEq/L), potassium 7.3 (mEq/L), lactic acid 10.6 millimole per liter (mmol/L), and anion gap 30. 

**Table 1:** Potassium, bicarbonate, and creatinine values

Time	Potassium (milliequivalents per liter (mEq/L))	Bicarbonate (milliequivalents per liter (mEq/L))	Creatinine (milligrams per deciliter (mg/dl))
On admission	7.4 (3.5-5.1)	5 (22-26)	7.3 (0.7-1.25)
After 2 hours (received hyperkalemia cocktails)	6.7 (3.5-5.1)	<5 (22-26)	7.7 (0.7-1.25)
After 5 hours of initiation of continuous renal replacement therapy (CRRT)	5.1 (3.5-5.1)	7 (22-26)	6.6 (0.7-1.25)
After 12 hours of CRRT	5.0 (3.5-5.1)	11 (22-26)	5.1 (0.7-1.25)
After 18 hours of CRRT	5.1 (3.5-5.1)	16 (22-26)	3.7 (0.7-1.25)
After 24 hours of CRRT	5.0 (3.5-5.1)	18 (22-26)	2.9 (0.7-1.25)
After 36 hours of CRRT	4.9 (3.5-5.1)	24 (22-26)	1.9 (0.7-1.25)
Day 2	5.0 (3.5-5.1)	19 (22-26)	1.7 (0.7-1.25)
Day 3 (stopped CRRT)	3.6 (3.5-5.1)	22 (22-26)	1.7 (0.7-1.25)
Day 4	3.6 (3.5-5.1)	20 (22-26)	2.4 (0.7-1.25)
Day 5	3.7 (3.5-5.1)	20 (22-26)	3.8 (0.7-1.25)
Day 6	3.6 (3.5-5.1)	20 (22-26)	4.9 (0.7-1.25)
Day 7	3.6 (3.5-5.1)	22 (22-26)	5.5 (0.7-1.25)
Day 8	3.7 (3.5-5.1)	22 (22-26)	5.4 (0.7-1.25)
Day 9	3.6 (3.5-5.1)	22 (22-26)	5.1 (0.7-1.25)
Day 10	4.2 (3.5-5.1)	22 (22-26)	4.9 (0.7-1.25)
Day 11	4.2 (3.5-5.1)	21 (22-26)	3.9 (0.7-1.25)
Day 12 (before discharge)	4.3 (3.5-5.1)	21 (22-26)	3.4 (0.7-1.25)

**Figure 1:**
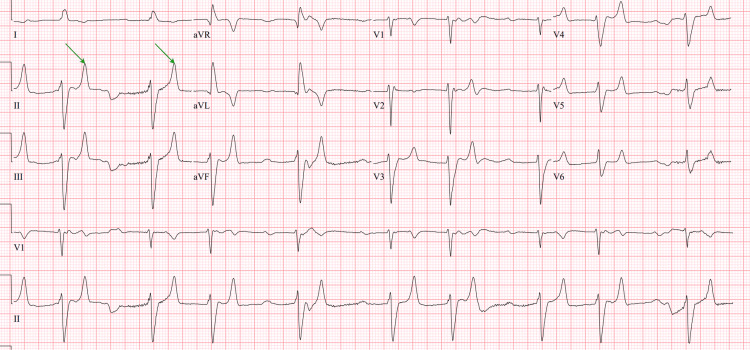
EKG with arrows showing tall and peaked T waves

**Figure 2:**
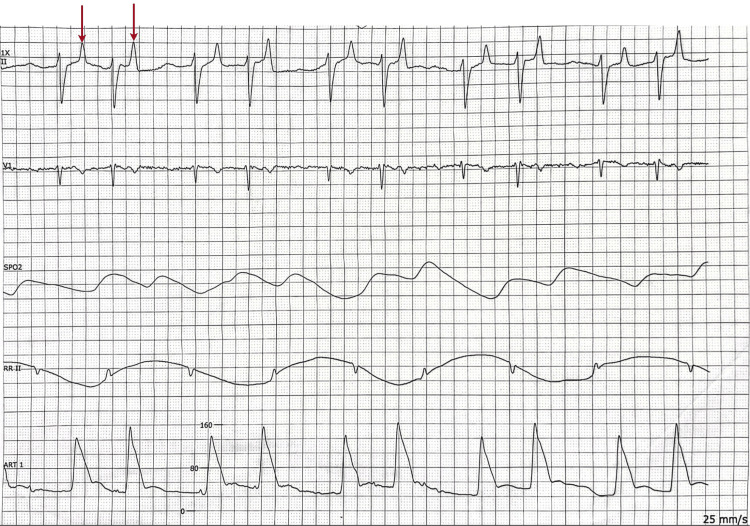
Telemetry strip with arrows showing tall and peaked T waves

**Figure 3:**
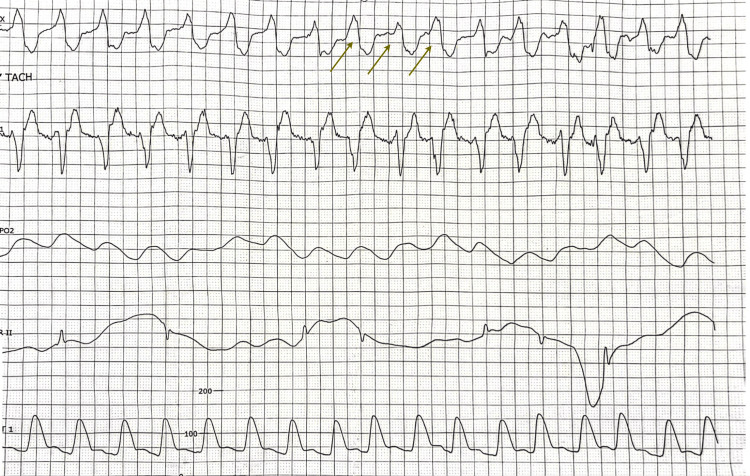
Telemetry strip with arrows showing widened QRS complexes

Given the presence of severe hyperkalemia with an electrocardiogram (EKG) showing a tall and peaked T wave (Figure 1), the patient received intravenous calcium gluconate and hyperkalemia cocktail containing insulin, albuterol nebulization, and sodium bicarbonate. Despite initial treatment, potassium levels remained elevated at 6.7 mEq/L and the patient developed a tall peak T wave (Figure 2) followed by wide complex QRS in telemetry (Figure 3) suggestive of impending cardiac arrest. The patient was given intravenous calcium gluconate and sodium bicarbonate. Consequently, he was intubated due to hemodynamic instability and started on continuous renal replacement therapy (CRRT) in the intensive care unit.

Potassium and lactate levels normalized after 6 hours and 36 hours of CRRT, respectively. The patient was successfully extubated after correction of hyperkalemia and acid-base status. Subsequent investigations for acute kidney injury superimposed on chronic kidney diseases, including complements C3, C4, multiple myeloma workup, anti-phospholipase A2 antibody, myeloperoxidase (p-ANCA) antibody, and serine protease 3 antibody (c-ANCA), came out negative. The patient’s spot protein to creatinine ratio was found to be 3.72 milligrams/milligram (mg/mg) with normal being less than <0.2 mg/mg, which suggests nephrotic range proteinuria. After extubation, CRRT was stopped and close monitoring of kidney function was done without the need for renal replacement therapy. Other tests done included computed tomography (CT) scans of the chest, abdomen, and pelvis, which showed no significant abnormality. An echocardiogram was done, which revealed a reduced ejection fraction of 30%, with hypokinesis of antero-apical myocardium. Coronary angiography confirmed complete total occlusion of the proximal left anterior descending artery (LAD) with minimal disease in other vessels.

Metformin levels were found to be elevated at 21 micrograms per milliliter (mcg/ml), with the normal therapeutic range being 1-2 mcg/ml. Hence, metformin was discontinued, and insulin detemir was prescribed for glycemic control. He was started on medical therapy for heart failure, with plans for further evaluation and consideration of interventions based on viability studies and symptom progression on an outpatient basis. He was discharged with appropriate medical management and plans for close follow-up with multiple specialties, including a nephrologist, cardiologist, and primary care physician.

## Discussion

Metformin is a well-tolerated drug favored as a first-line treatment for the management of diabetes mellitus owing to its safety, tolerability, and possible cardiovascular benefits [1]. Metformin belongs to a class of drugs called biguanides whose other members phenformin and buformin were withdrawn from the market due to evidence linking increased risk of severe lactic acidosis with their use. There are multiple case reports and case series that record metformin-associated lactic acidosis (MALA) in patients of DM2 under metformin treatment [2].

The mechanism of action of metformin on a molecular level is inhibition of complex I of the respiratory chain, which leads to activation of adenosine monophosphate-activated protein kinase (AMPK) in hepatocytes, leading to an inhibition of hepatic gluconeogenesis and thus lowering the blood glucose level [4]. The inhibition of complex I in the hepatocytes by metformin leads to the inhibition of mitochondrial oxidative phosphorylation, which can potentially cause an imbalance between overall lactate production and hepatic metabolism of lactate, especially in conditions like sepsis resulting in lactic acidosis [4,5]. Metabolic acidosis can result in hyperkalemia through the inhibition of the sodium-potassium adenosine triphosphate (ATPase) pump. This results in a seeming entry of a proton into the cell in exchange for potassium being pumped out of the cell [6]. Although hyperkalemia is a more frequent occurrence in hyperchloremic metabolic acidosis (mineral acidosis), it is also observed in organic acidosis like ketoacidosis and lactic acidosis [7,8]. This chain of events is a possible explanation as to how MALA, and thus metformin, can cause hyperkalemia.

According to a retrospective cohort study, the crude incidence of lactic acidosis or elevated lactate levels (>5 mmol/L) was 7.4 per 100,000 person-years in current metformin users compared to 2.2 per 100,000 person-years in non-users. This risk was significantly associated with a renal function of <60 mL/min/1.73 m^2^ [9]. However, a systematic review that included 347 comparative trials and cohort studies did not find any increased risk of lactic acidosis nor any difference in the plasma lactate levels between the metformin and non-metformin-treated groups [10]. Even though MALA is a rare condition, the condition is life-threatening with a mortality rate of 30% [11].

As metformin is predominantly excreted by the kidneys, a fear of accumulation of metformin leading to lactic acidosis in patients with CKD leads to guidelines restricting the use of metformin in patients with a creatinine level of more than 1.4 mg/dl [12]. However, due to newer evidence [13], the United States Foods and Drug Administration (US FDA) recently updated its guidelines, which allow the use of metformin in mild to moderate renal impairment. The guideline does not recommend initiation but allows for the continuation of metformin in patients with an estimated glomerular filtration rate (eGFR) from 30-45 mL/min/1.73 m^2^ [14]. Our patient had CKD stage 3A (GFR 30-45 mL/min/1.73 m^2^) and he was continued on his metformin dosage for DM2. A single-center study recommended dosages of 1500 mg, 1000 mg, and 500 mg in patients with CKD stage 3A, 3B, and 4, respectively, as the serum metformin concentration and serum lactate levels never exceeded the safe upper limit of normal even after four months of therapy [15]. Our patient with CKD stage 3A was on a daily regimen of 1500 mg of metformin, which is acceptable as per his kidney function.

In our case, the patient presented with complaints of poor appetite and diarrhea for two to three days. His baseline creatinine of 1.7 mg/dl measured two months back had increased to 8 mg/dl, suggesting acute renal dysfunction on a pre-existing chronic kidney disease, which seems to have triggered metformin toxicity resulting in severe metabolic acidosis and hyperlactatemia with the lactate levels rising to 14.9 mmol/L. In patients with CKD taking metformin, acute gastroenteritis leading to dehydration triggering acute renal dysfunction is a common cause of MALA [16]. In our case, the cause of the acute renal dysfunction was multifactorial due to acute gastroenteritis, poor oral intake, and urinary tract infection.

The severe hyperkalemia(K=7.8) in our case may be due to variable etiology with pre-existing chronic kidney disease and acute renal dysfunction being the usual suspects. Although treatment with metformin is associated with higher potassium levels, this effect has been attributed to the underlying diabetes mellitus rather than the effect of metformin per se [17]. However, MALA, which resulted from metformin toxicity, could have been an important trigger for hyperkalemia in our case. Important mechanisms by which diabetes predisposes to hyperkalemia are prevention of dispersion of potassium into the intracellular space, paradoxical rise in potassium levels induced by hyperglycemia, hyporeninemic hypoaldosteronism, and decreased aldosterone production despite normal renin secretion [17,18]. In a nested case-control study by Loutradis et al., diabetes mellitus was found to elevate the incidence of hyperkalemia in patients with CKD stage 3 (moderately impaired renal function) [19]. In our case, hyperkalemia was most probably due to CKD with superimposed acute renal dysfunction with diabetes mellitus and MALA being important contributing factors.

In a case series of 29 patients with metformin toxicity associated with acute kidney injury and lactic acidosis, 59% had baseline CKD (28% stage 3a and 31% stage 3b). Fifty-five percent (55%) of the patients required continuous renal replacement therapy (CRRT) and 52% were admitted to the ICU [16]. Early CRRT is a safe and effective method for managing MALA, especially in hemodynamically unstable patients who are unable to tolerate intermittent hemodialysis [20]. In our case, the patient was admitted to the ICU. He was hemodynamically unstable requiring vasopressors and thus was initiated on early CRRT after which his metabolic parameters improved.

## Conclusions

Metformin-associated lactic acidosis (MALA) is a rare but potentially fatal condition that can occur in patients with chronic kidney disease (CKD) taking metformin. Despite guidelines, MALA can still occur, and hyperkalemia can coexist with it. Physicians should be vigilant for hyperkalemia in MALA patients and act promptly based on laboratory values and electrocardiogram (ECG) readings to prevent severe consequences.

This case emphasizes the need for early recognition and prompt discontinuation of metformin, along with appropriate management of electrolyte disturbances and metabolic derangements, to prevent life-threatening complications. It also underscores the importance of close monitoring of renal function in patients receiving metformin therapy, particularly in those with underlying renal impairment, especially in primary settings. This case also serves as a reminder to the physician to consider metformin toxicity in the differential diagnosis of severe hyperkalemia and metabolic acidosis in patients with AKI or CKD.

## References

[1] Inzucchi SE (2017). Is it time to change the type 2 diabetes treatment paradigm? No! Metformin should remain the foundation therapy for type 2 diabetes. Diabetes Care.

[2] Juneja D, Nasa P, Jain R (2022). Metformin toxicity: a meta-summary of case reports. World J Diabetes.

[3] Einhorn LM, Zhan M, Hsu VD (2009). The frequency of hyperkalemia and its significance in chronic kidney disease. Arch Intern Med.

[4] Rena G, Hardie DG, Pearson ER (2017). The mechanisms of action of metformin. Diabetologia.

[5] DeFronzo R, Fleming GA, Chen K, Bicsak TA (2016). Metformin-associated lactic acidosis: current perspectives on causes and risk. Metabolism.

[6] Aronson PS, Giebisch G (2011). Effects of pH on potassium. New explanations for old observations. J Am Soc Nephrol.

[7] Oster JR, Perez GO, Vaamonde CA (1978). Relationship between blood pH and potassium and phosphorus during acute metabolic acidosis. Am J Physiol.

[8] Lee Hamm L, Hering-Smith KS, Nakhoul NL (2013). Acid-base and potassium homeostasis. Semin Nephrol.

[9] Eppenga WL, Lalmohamed A, Geerts AF (2014). Risk of lactic acidosis or elevated lactate concentrations in metformin users with renal impairment: a population-based cohort study. Diabetes Care.

[10] Salpeter SR, Greyber E, Pasternak GA, Salpeter Posthumous EE (2010). Risk of fatal and nonfatal lactic acidosis with metformin use in type 2 diabetes mellitus. Cochrane Database Syst Rev.

[11] Peters N, Jay N, Barraud D, Cravoisy A, Nace L, Bollaert PE, Gibot S (2008). Metformin-associated lactic acidosis in an intensive care unit. Crit Care.

[12] Sulkin TV, Bosman D, Krentz AJ (1997). Contraindications to metformin therapy in patients with NIDDM. Diabetes Care.

[13] Vasisht KP, Chen SC, Peng Y, Bakris GL (2010). Limitations of metformin use in patients with kidney disease: are they warranted?. Diabetes Obes Metab.

[14] (2017). FDA Drug Safety Communication: FDA revises warnings regarding use of the diabetes medicine metformin in certain patients with reduced kidney function. https://www.fda.gov/drugs/drug-safety-and-availability/fda-drug-safety-communication-fda-revises-warnings-regarding-use-diabetes-medicine-metformin-certain#:~:text=Before%20starting%20metformin%2C%20obtain%20the,in%20all%20patients%20taking%20metformin..

[15] Lalau JD, Kajbaf F, Bennis Y, Hurtel-Lemaire AS, Belpaire F, De Broe ME (2018). Metformin treatment in patients with type 2 diabetes and chronic kidney disease stages 3A, 3B, or 4. Diabetes Care.

[16] Arroyo D, Melero R, Panizo N (2011). Metformin-associated acute kidney injury and lactic acidosis. Int J Nephrol.

[17] Belmar Vega L, Galabia ER, Bada Da Silva J (2019). Epidemiology of hyperkalemia in chronic kidney disease. Nefrol Engl Ed.

[18] Uribarri J, Oh MS, Carroll HJ (1990). Hyperkalemia in diabetes mellitus. J Diabet Complications.

[19] Loutradis C, Tolika P, Skodra A, Avdelidou A, Sarafidis PA (2015). Prevalence of hyperkalemia in diabetic and non-diabetic patients with chronic kidney disease: a nested case-control study. Am J Nephrol.

[20] Keller G, Cour M, Hernu R, Illinger J, Robert D, Argaud L (2011). Management of metformin-associated lactic acidosis by continuous renal replacement therapy. PLoS One.

